# Laparoscopic pyeloplasty in neonates and infants is safe and efficient

**DOI:** 10.3389/fped.2024.1397614

**Published:** 2024-07-26

**Authors:** S. Langreen, B. Ludwikowski, J. Dingemann, B. M. Ure, A. D. Hofmann, J. F. Kuebler

**Affiliations:** ^1^ Pediatric Surgery Clinic, Center for Pediatrics and Adolescent Medicine, Hannover Medical School, Hanover, Germany; ^2^ Department of Pediatric Surgery, Kinder- und Jugendkrankenhaus AUF DER BULT, Hanover, Germany

**Keywords:** laparoscopic pyeloplasty, laparoscopy in infants, ureteropelvic junction obstruction (UPJO), laparoscopic pyeloplasty in infant and neonates, pyeloplasty pediatrics, UPJO infants, UPJO neonates

## Abstract

**Introduction:**

Dismembered laparoscopic pyeloplasty (LP) is a well-accepted treatment modality for ureteropelvic junction obstruction (UPJO) in children. However, its efficacy and safety in infants, particularly neonates, remain uncertain. To address this significant knowledge gap, we aimed to compare outcomes between a cohort of neonates and infants undergoing LP vs. open pyeloplasty (OP) at less than 6 months and 6 weeks of age.

**Material and methods:**

We conducted a retrospective analysis of data from patients who underwent primary pyeloplasty at our institution between 2000 and 2022. Only patients aged 6 months or less at the time of surgery were included, excluding redo-procedures or conversions. Ethical approval was obtained, and data were assessed for redo-pyeloplasty and postoperative complications, classified according to the Clavien–Madadi classification. A standard postoperative assessment was performed 6 weeks postoperatively. This included an isotope scan and a routine ultrasound up to the year 2020.

**Results:**

A total of 91 eligible patients were identified, of which 49 underwent LP and 42 underwent OP. Patients receiving LP had a median age of 11.4 (1–25.4) weeks, compared to 13.8 (0.5–25.9) weeks for those receiving OP (*p* > 0.31). Both groups in our main cohort had an age range of 0–6 months at the time of surgery. Nineteen patients were younger than 6 weeks at the time of surgery. The mean operating time was longer for LP (161 ± 43 min) than that for OP (109 ± 32 min, *p* < 0.001). However, the mean operating time was not longer in the patient group receiving LP at ≤6 weeks (145 ± 21.6) compared to that in our main cohort receiving LP. There was no significant difference in the length of stay between the groups. Four patients after LP required emergency nephrostomy compared to one patient after OP. The rate of revision pyeloplasty in our main cohort aged 0–6 months at surgery was 8% in the patient group receiving LP and 14% in the patient group receiving OP (not significant). Three revisions after LP were due to persistent UPJO, and one was due to stent migration. Only one patient requiring revision pyeloplasty was less than 6 weeks old.

**Conclusion:**

To our knowledge, this is one of the largest collectives of laparoscopic pyeloplasty performed in infants, and it is the youngest cohort published to date. Based on our experience, LP in neonates and infants under 6 months appears to be as effective as open surgery.

## 1 Introduction

Ureteropelvic junction obstruction (UPJO) is a common cause of hydronephrosis in children, resulting in renal damage if not treated in a timely fashion (1). The treatment of choice is dismembered pyeloplasty (2, 3). In addition to conventional open pyeloplasty (OP), minimally invasive pyeloplasty (MIP) has become a widely accepted treatment modality in children, and it offers benefits such as shorter hospital stays and superior cosmetic results (4, 5). However, performing minimally invasive surgery in infants is technically challenging (6–9). Therefore, conventional pyeloplasty remains the most frequently utilized approach, accounting for over 80% of all pyeloplasties in the United States (10). In robotic-assisted laparoscopic pyeloplasty (RALP), with camera port sizes of 8.5 mm and work port sizes of a minimum of 5 mm, patient age and weight are strong limitations (10). Thus, despite it being the preferred minimal invasive approach in older children in the United States (US) (7), infants below 1 year are 40 times less likely to receive robotically assisted pyeloplasty than older children (4). Apart from one report from Beijing (11), the published robotic series do not include patients below 3 months of age (12–14).

Metzelder et al. (15) from our department have demonstrated the feasibility of laparoscopic pyeloplasty (LP) in children irrespective of their age. Subsequent studies have further attested to the feasibility of LP in infants younger than 1 year (16, 17) and 6 months (18), showing similar results to that in open surgery. As a result, we have shifted our treatment of choice from open to laparoscopic pyeloplasty across all age groups.

Despite these advancements, specific evidence regarding the efficacy and safety of LP in neonates remains limited. Therefore, we retrospectively analyzed the results of laparoscopic pyeloplasties in infants (≤6 months) and neonates (≤6 weeks) to address the significant knowledge gap in the literature.

## 2 Patients and methods

### 2.1 Patient collective

A retrospective data analysis was performed on all patients who received dismembered pyeloplasty in our department from 2000 until 2022 and were aged 6 months or younger. Ethical approval was obtained from the ethics committee at Hannover Medical School (no. 10331_NO_K2022).

All patients received preoperative sonography and renal isotope scan for establishment of diagnosis. The surgical treatment of choice was either laparoscopic pyeloplasty (LP) or open pyeloplasty. Patients were retrospectively divided into two groups according to the utilized surgical approach. Group OP (OP) comprised patients who underwent open pyeloplasty, and Group LP comprised patients who underwent laparoscopic treatment.

Additionally, out of this collective, another subgroup including only the patients younger than 6 weeks was analyzed, thereby comparing the LP vs. OP at 0–6 months.

Three patients of this collective have been reported in a previous retrospective case series, and none of them were under 6 weeks of age at surgery (15).

Indication for surgery was identical for all patients independent of the chosen approach: significant obstruction in renal isotope scan, defined as <50% clearance after 30 min, furosemide application, and voiding. Furthermore, an increase in hydronephrosis in combination with either a decrease in renal split function (<40%) or borderline isotope scan would lead to surgical treatment, as equally the evidence of symptoms would. The decision to perform open or laparoscopic surgery was based on the surgeon’s preference.

### 2.2 Surgical technique

Pyeloplasty was performed according to a standardized method as previously published (15).

In the open approach, the incision was made lumbar subcostal or transverse abdominal, and the retroperitoneal space was exposed by blunt dissection. After identification of the ureter, further dissection cranially led to the obstructed ureter–pelvic junction. The kidney and ureter were mobilized to optimize exposure, with fine-holding sutures positioned at the proximal ureter. After transection of the ureter and renal pelvis with the removal of the obstructed part, Anderson–Hynes anastomosis was performed with Vicryl 5–0 or 6–0 interrupted sutures.

For the laparoscopic approach, 5 mm or 3.5 mm trocars were used for camera access, and two more 3.5 mm trocars were positioned in a triangular fashion. Either the colic flexure was mobilized, or a transmesenteric approach was chosen to expose the renal pelvis and identify the ureteral junction obstruction. In some patients, the renal pelvis was elevated by transcutaneous traction sutures for better exposure.

After the dissection of the ureter with the removal of the obstructive part, the ureter was incised and spatulated longitudinally. Pyeloureteric anastomosis was performed with Vicryl interrupted or continuous sutures (6–0 or 5–0) without major reduction of the pelvis (19).

A transanastomotic nephrostomy catheter or double J catheter was routinely placed before the completion of pyeloplasty. The former remained in position for 7–10 days, while for the latter, removal by cystoscopy after 2–4 weeks was performed (20, 21). Antibiotic therapy was administered until the catheter was removed.

### 2.3 Follow-up

A standard postoperative assessment was conducted 6 weeks postoperatively in our outpatient clinic. This included a routine ultrasound and an isotope scan up to the year 2020. Following the publication of Kiblawi et al. in 2020, which demonstrated the high sensitivity of ultrasound monitoring after pelvis-sparing pyeloplasty, the follow-up protocol was modified (22). Subsequently, reduction of renal pelvis diameter or the maintenance of a stable diameter no longer prompted an isotope scan. Initiation of the renal scan was reserved for patients demonstrating an increase in anteroposterior diameter or patients with symptoms such as pain or recurring urinary tract infections.

### 2.4 Endpoints

Successful pyeloplasty was defined as a primary endpoint. Surgery was marked successful when there was no need for reoperation and sufficient urine drainage was detected either through renal isotope scan (at least 50% clearance after 30 min, furosemide application and voiding, stable split function) or ultrasound showing stable or decreasing AP diameter.

Furthermore, we compared the incidence of postoperative complications, operating times, and length of hospital stay. As part of our in-house protocol, complications were graded according to the Clavien–Madadi criteria on a daily basis. This classification system—based on the Clavien–Dindo criteria—was designed specifically for assessing surgical complications in pediatric patients. Those requiring any type of surgical intervention under general anesthesia were categorized as either grade IIIa or IIIb, with the distinction being laparotomy for grade IIIb and any endoscopic, radiologic, or laparoscopic intervention for grade IIIa (22–24).

### 2.5 Statistical analysis

Numerical data is presented either as median alongside its range or as mean with standard deviation (±).

We performed a Student's *t*-test to determine significant differences between two normally distributed groups for quantitative variables and a chi-squared test or Fisher's exact test for qualitative data. The confidence interval was set at 95%.

## 3 Results

### 3.1 Patient demographics

#### 3.1.1 0–6 months

Ninety-three primary pyeloplasties were performed on patients younger than 6 months in our department of pediatric surgery in the last 20 years. A primary laparoscopic approach was chosen in 51 patients. In two patients, conversion to open surgery was required, due to technical difficulties, leading to 49 performed laparoscopic pyeloplasties. Out of the 44 patients receiving open pyeloplasty, only the 42 patients who had primarily been planned for open surgery were included in this report.

There was no significant difference in weight or age of the groups. Similarly, there were no significant differences in the severity of preoperative hydronephrosis, the necessity for an urgent postnatal nephrostomy, or the duration of follow-up (Table 1).

**Table 1:** Patient demographics (main cohort age of 0–6 months); the LP group comprised patients receiving laparoscopic pyeloplasty, and the OP group comprised patients receiving open pyeloplasty.

*** ***	LP group *n* = 49	OP group *n* = 42	*p*-value
Age (weeks)	11.4 (1–25.4)	13.8 (0.5–25.9)	0.31
Weight at surgery (g)	5,791 ± 1,686	5,425 ± 1,771	0.32
Hydronephrosis Grade IV	26 (53%)	20 (48%)	0.97
Postnatal nephrostomy	6 (12%)	8 (19%)	0.99
Follow-up (months)	27 ± 22.5	45.4 ± 45	**0.025**

Statistically significant *p*-values are in bold.

**Figure 1:**
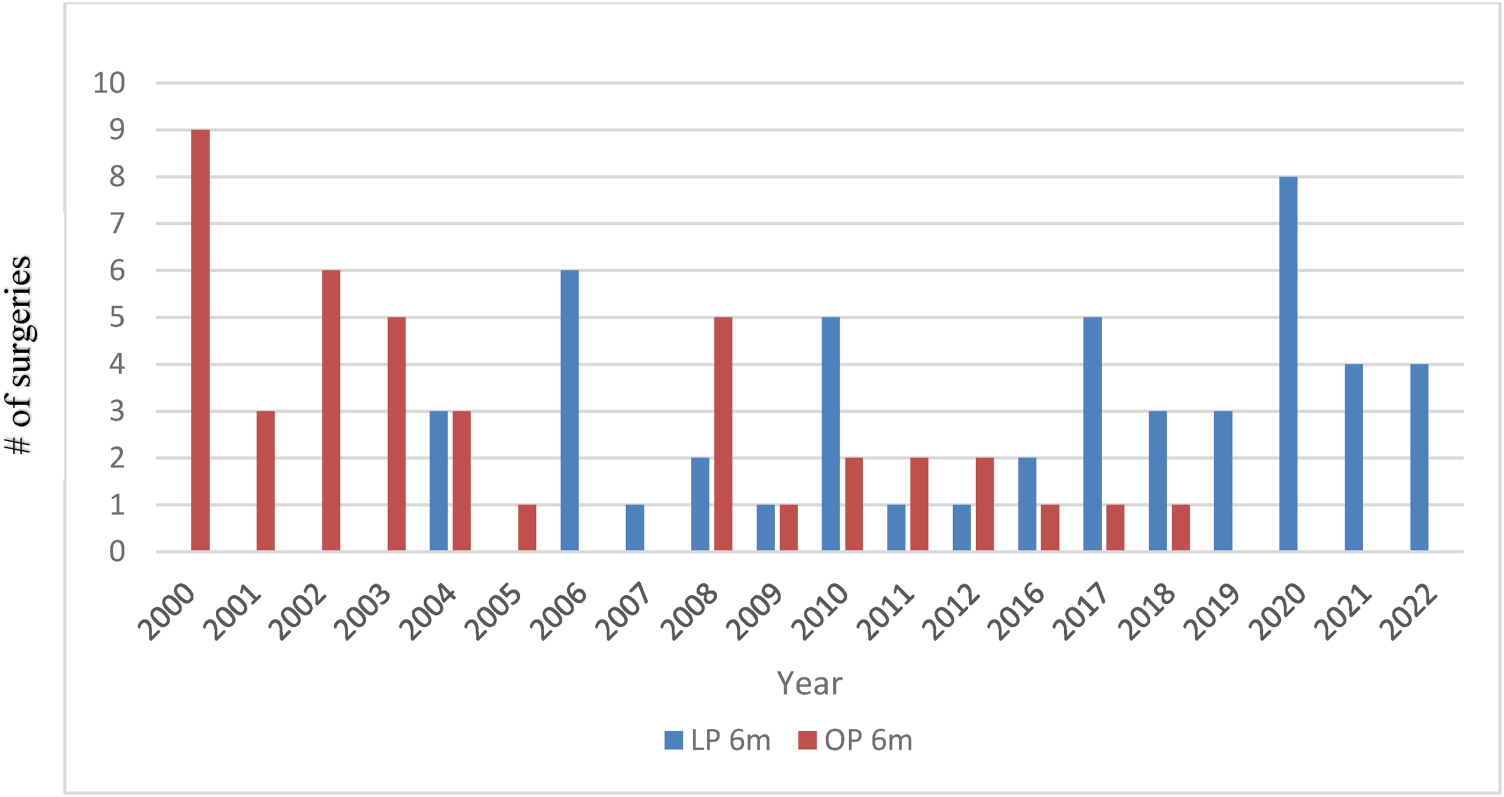
Distribution of open (OP) vs. laparoscopic (LP) approach in patients aged ≤6 months over the years.

In the early years of this report, an open approach was chosen in all patients aged less than 6 months. However, this has changed over the years, wherein current practice now involves routine laparoscopy in this patient cohort with an evident steady decrease in OP. Since 2019, all primary Anderson–Hynes surgeries in patients less than 6 months were performed laparoscopically (Figure 1).

#### 3.1.2 0–6 weeks

Additionally, we analyzed a subgroup only including all patients who had undergone surgery at 6 weeks of age or younger, within our main collective. Out of 19 patients receiving pyeloplasties, a laparoscopic approach was used in 9, and an open approach was used in 10 patients.

In this subgroup, the median age at surgery was 3.4 weeks (range, 1–5 weeks) in patients receiving LP and 5.1 weeks (range, 2–6 weeks) for those receiving OP. The mean weight was 3,416 g ± 633 and 3,833 g ± 758, respectively. Except for the follow-up (24.1 ± 10.4 months in the group receiving LP and 69.4 ± 42.7 months in the group receiving OP), no significant difference between the groups was detected (Table 2).

**Table 2:** Patient demographics (subcohort age of 0–6 weeks); the LP group comprised patients receiving laparoscopic pyeloplasty, and the OP group comprised patients receiving open pyeloplasty.

*** ***	LP ≤6 weeks *n* = 9	OP ≤6 weeks *n* = 10	*p*-value
Age (weeks)	3.4 (1–5)	5.2 (2–6)	0.076
Weight at surgery (g)	3,416 g ± 633	3,833 ± 758	0.25
Hydronephrosis Grade IV	9 (100%)	6 (60%)	0.83
Postnatal nephrostomy	3 (33%)	3 (30%)	0.74
Follow-up (months)	24.1 ± 10.4	69.4 ± 42.7	**0.01**

Statistically significant *p*-values are in bold.

### 3.2 Perioperative data

#### 3.2.1 0–6 months

The mean operating time was significantly longer in laparoscopic surgery, 161 ± 43 as opposed to 109 min ± 32 in open surgery (*p* < 0.001).

No significant difference regarding the days of hospitalization was detected (Table 3).

**Table 3:** Perioperative data (main cohort age of 0–6 months); the LP group comprised patients receiving laparoscopic pyeloplasty, and the OP group comprised patients receiving open pyeloplasty.

* *	LP group *n* = 49	OP group *n* = 42	*p*-value
Surgery time (minutes)	161 ± 43	109 ± 32	**<0** ***.*** **001**
Hospitalization (days)	7.3 ± 3.7	9.7 ± 6	0*.*03
Conversion	2 out of 51 (4%)		

Statistically significant *p*-values are in bold.

#### 3.2.2 0–6 weeks

Significantly longer operating times were also seen in the group receiving LP in the under-6-week subgroup, 145 ± 21.6 compared to 95 min ± 28.8 in the group receiving OP (*p* < 0.001). Again, there was no significant difference regarding the days of hospitalization (Table 4).

**Table 4:** Perioperative data (subcohort age of 0–6 weeks); the LP group comprised patients receiving laparoscopic pyeloplasty, and the OP group comprised patients receiving open pyeloplasty.

* *	LP ≤6 weeks *n* = 9	OP ≤6 weeks *n* = 10	*p*-value
Surgery time (minutes)	145 ± 21.6	95 ± 28.8	**<0** ***.*** **001**
Hospitalization (days)	10.4 ± 4.3	10.2 ± 276	0*.*88

Statistically significant *p*-values are in bold.

### 3.3 Complication rates

#### 3.3.1 0–6 months

Emergency postoperative nephrostomy was required in four patients within the group after LP (8%) and in one case within the group after OP (2%). In the latter, a nephrostomy was necessary due to inadequate renal drainage. Consecutively leading to ureteropelvic junction revision. A similar cause was noted in one of the four patients after LP.

Nephrostomy placement indication included severe persistence or recurrence of hydronephrosis with clinical symptoms (abdominal pain, vomiting, or increased serum creatinine) or with evident obstruction in the postop isotope scan, regardless of symptoms.

Redo-pyeloplasty was performed in four patients (8%) after LP and in six patients (14%) after OP. After LP, three patients required revisions (6%) due to persistent/recurrent obstruction. The other patient (2%) received emergency revision within 48 h after an accidental stent dislocation and subsequent anastomotic insufficiency with leakage. Revision pyeloplasty in the group after LP was performed laparoscopically in two of four patients, while all of the six patients, needing revision after OP received an open approach.

Two patients (4%) developed early postoperative fever after OP, one due to gastrointestinal infection and one due to urinary tract infection (UTI). One of the patients after LP developed a fever due to UTI, and this patient received nephrostomy in addition to antibiotic therapy due to inflammation-induced urinary stasis that resolved after treatment (Table 5).

**Table 5:** Complications (main cohort age of 0–6 months); the LP group comprised patients receiving laparoscopic pyeloplasty, and the OP group comprised patients receiving open pyeloplasty.

* *	LP group *n* = 49	OP group *n* = 42	*p*-value
Nephrostomy	4 (8%)	1 (2%)	0*.*95
UPJO revision	4 (8%)	6 (14%)	0*.*99
Fever	1 (2%)	2 (4%)	0*.*99

Complications underwent grading based on the Clavien–Madadi criteria for pediatric patients.

No patient suffered from major adverse events (Grade IV or V Clavien–Madadi criteria) such as bleeding, sepsis, perforation of the bowel, or death in either group. No significant difference could be detected between any of the groups (Table 6).

**Table 6:** Complications (main cohort age of 0–6 months) according to the Clavien–Madadi criteria; the LP group comprised patients receiving laparoscopic pyeloplasty, and the OP group comprised patients receiving open pyeloplasty.

Clavien–Madadi criteria	LP group *n* = 49	OP group *n* = 42	*p*-value
I a	0	0	
I b	0	1 (2%)	0.46
II	0	1 (2%)	0.46
IIIa	5 (12%)	0	0.04
III b	2 (4%)	6 (14%)	0.07
IV	0	0	
V	0	0	

#### 3.3.2 0–6 weeks

Of all the patients after LP ≤6 weeks, only one (11%) required a pyeloplasty revision. The same patient had undergone a previous nephrostomy. Of all the patients after OP, two (20%) underwent revision pyeloplasty. No statistically significant difference was observed between the two groups (Table 7).

**Table 7:** Complications (subcohort age of 0 6 weeks); the LP group comprised patients receiving laparoscopic pyeloplasty, and the OP group comprised patients receiving open pyeloplasty.

* *	LP ≤6 weeks *n* = 9	OP ≤6 weeks *n* = 10	*p*-value
Nephrostomy	1 (11%)	0	0*.*47
UPJO persistence	1 (11%)	2 (20%)	0*.*42

## 4 Discussion

Laparoscopic pyeloplasty has already been proven feasible and safe in the pediatric population, even in infants younger than a year (2, 15–17, 25) and under 6 months of age (18).

Metzelder et al. (15) from our department established that a laparoscopic approach yields good outcomes in children under one year. This has led to a gradual shift in the treatment of choice from open to laparoscopic approach in all patients over the years.

However, to our knowledge up to now, the youngest patient reported to undergo laparoscopic pyeloplasty was 6 weeks old. Kutikov et al. have published a series of a patient collective at a mean age of 4.5 months (range, 3–5 months) (18). We aimed to investigate whether there are any problems with the laparoscopic approach in very young infants.

Even though our general study cohort included patients younger than 6 months, approximately 20% of these patients were less than 6 weeks old at surgical treatment. Therefore, to our knowledge, this group is one of the youngest patient series ever reported (mean age, 2.6 months; youngest age, 1 week) receiving minimally invasive pyeloplasty. This is also reflected in the weight distribution of our study group, the smallest patient weighing 2,600 g at the time of laparoscopic surgery (mean weight of 5,791 g), compared to the lowest weight disclosed in previous publications (minimum weight of 4,000 g) (15).

Arguably, the technical challenges of laparoscopic intraabdominal suturing are amplified by the restricted operative space in small patients, possibly leading to longer surgical time and greater risks in neonates and infants (26). However, even though operating time was significantly increased in the group of patients receiving laparoscopy compared to the patients receiving an open approach, our results matched the range of previously published reports for laparoscopic (16, 27) and robotic approaches in older children (12, 28, 29). More importantly, the subgroup younger than 6 weeks did not require longer surgical time than the older patients undergoing laparoscopic surgery. (Mean surgical time of 161 min in the overall group receiving LP vs. 145 min in the subgroup receiving LP at less than 6 weeks). Hence, supporting the thesis, that younger age does not exert a negative influence on surgery time and feasibility. The majority of the patients younger than 6 weeks presented with severe hydronephrosis (100% SFU Grade IV hydronephrosis in the group receiving LP, 60% in the group receiving OP). In cases of acute progression, an increase of serum creatinine levels, or a history of intrauterine amniopelvic splint implantation, placement of urgent postnatal nephrostomy was indicated (23% in the LP group ≤6 weeks; 30% in the OP group, ≤6 weeks). Unfortunately, we observed an extensive level of pelvic tissue inflammation in these patients, which was not present in patients without renal intervention prior to surgery, consequently resulting in more challenging conditions during Anderson–Hynes surgery. In these selected patients, foregoing preoperative renal decompression and performing direct pyeloplasty would prevent another anesthesia and avoid possible complications such as dislocation, bleeding, and infection. Furthermore, surgical conditions would be optimized.

The most important factor in the assessment of efficacy was recurrent obstruction. Our case series did not reveal a higher rate of recurrence or persistence in the group of patients receiving LP.

Pyeloplasty revision rates after the laparoscopic approach have been reported between 0% and 17% (12, 17, 25, 28, 29). This is congruent to our results in the main group with UPJO revision in 8% of the patients (6% due to persistent obstruction, 2% due to anastomotic leakage). In the 6-week subgroup, only one patient required revision surgery for obstruction, leading to an overall recurrence percentage of 11%.

Comparing hospitalization time in our patient cohort, we generally advocate for early discharge of patients after LP with indwelling nephrostomy catheter, irrespective of the patient’s age. However, given the very young age of this patient group, parenteral insecurity about catheter handling often resulted in longer hospitalization time. This confounding factor calls into question the suitability of hospitalization time as a representative measure.

Data analysis was retrospective, and therefore follow-up protocol was not identical for all patients.

Following the publication of Kiblawi et al. in 2020 (30), an isotope scan was no longer a routinely performed 6 weeks postsurgery, unless an increase in AP diameter became evident during the 6-week follow-up ultrasound or the patient exhibited symptoms (pain or recurring UTIs).

Considering the study design, the presence of a selection bias between the two groups cannot completely be ruled out, thereby limiting their comparability. Nonetheless, this should not diminish the positive results observed in the group of patients receiving LP.

Our overall results in the group of patients receiving LP at under 6 months and under 6 weeks are promising in comparison to open and robotic-assisted pyeloplasty. However, one should keep in mind that laparoscopic pyeloplasty in this age group was only performed by pediatric surgery fellows or consultants and not by young residents. The laparoscopic technique, in our opinion, is technically more challenging than the open procedure.

## 5 Conclusion

This is to our knowledge one of the largest collectives of laparoscopic and pyeloplasty in infants, including the youngest patients published so far. Based on experience, we consider laparoscopic pyeloplasty in neonates and infants under 6 months as least as effective as open surgery.

## Data Availability

The raw data supporting the conclusions of this article will be made available by the authors, without undue reservation.
